# Development and Validation of a Novel Holistic Skin Quality Assessment Scale

**DOI:** 10.1111/jocd.16615

**Published:** 2024-10-09

**Authors:** Christoph Martschin, Ruba Bahhady, Jason Li, Walter Loureiro, Wesam Mansour, Andrei Metelitsa, Kuldeep Minocha, Michael Somenek, Keywan Taghetchian, Tanongkiet Tienthavorn

**Affiliations:** ^1^ Private Practice Lisbon Portugal; ^2^ Premium Cosmetic Laser Center Dubai UAE; ^3^ JFM Dermatology Clinic Taipei Taiwan; ^4^ Department of Dermatology University of the State of Pará Belém Brazil; ^5^ New Look Medical Center Al Ain UAE; ^6^ Beacon Dermatology Calgary Alberta Canada; ^7^ L'ART by Dr M London UK; ^8^ Somenek+PittmanMD Washington District of Columbia USA; ^9^ Clinic Smoothline Munich Germany; ^10^ Clinic Smoothline Zurich Switzerland; ^11^ Division of Dermatosurgery Institute of Dermatology, Ministry of Public Health Bangkok Thailand

**Keywords:** physician, skin quality assessment scale, subject, tool

## Abstract

**Background:**

Radiant skin is a common patient request and the result of multiple contributing factors. Currently, there is no standardized methodological approach that facilitates assessment of skin quality from a holistic perspective.

**Aim:**

To develop a holistic methodological process to assess skin quality using a scale that helps identify treatment priorities, facilitates conversation with the subject, and helps manage expectations, supports long‐term treatment plans, and tracks treatment progress over time.

**Methods:**

Ten global experts (dermatologists and esthetic physicians) identified the main measurable aspects that contribute to skin quality, and these were combined to form the Skin Quality Assessment Scale (SQS). The scale comprises four overarching skin quality domains containing nine measurable aspects: texture (pores, lines, scars); discoloration (redness, pigmentation, dullness); firmness (laxity); and hydro‐lipid balance (oiliness, dryness). Each aspect is graded on a 4‐point severity scale (0 = none to 3 = severe). The SQS was validated by a large group of practicing clinicians.

**Results:**

Practicing clinicians (> 40, 78% dermatologists) were surveyed; prior to reviewing the scale, 67% did not use any scale but 81% believed a holistic SQS was needed. After reviewing the scale, 100% agreed the scale provides a holistic assessment of skin quality. In addition, 95% agreed the scale helps assess all key aspects of skin quality with subjects and 98% deemed it valuable for their clinic.

**Conclusions:**

The SQS represents a holistic assessment tool that engages with and manages subjects' expectations, identifies treatment priorities, creates a long‐term treatment plan, and visualizes the skin quality improvement over time.

## 1 Introduction

The visible condition of the skin and its quality contribute to facial attractiveness [1, 2, 3, 4] and also influence the perception of age and health [2, 3, 5]. As a result, skin quality has a major impact on emotional and mental health, quality of life, self‐perception, and social interactions [6, 7, 8, 9]. This holds true globally for males and females and across all ethnicities [3, 4, 10, 11], and while specific aspects, such as the preference of skin tone, differ from east to west [12, 13, 14, 15, 16, 17], all strive to achieve the common goal of healthy, youthful‐looking, flawless skin [18, 19, 20]. The focus on flawless, glowing skin has further intensified over recent years as a result of the constant confrontation with our close‐up appearance via social media, online meetings, and video calls.

Multiple factors contribute to skin quality and existing scales focus on specific aspects, such as acne scars [21], rosacea [22], skin texture, and homogenous pigmentation [1]. In recent publications focusing on the multifaceted nature of skin quality, it was highlighted that currently, no integrated holistic skin quality assessment scale exists and that there is a clear need for a standardized methodological approach [8, 23].

To fulfill the need for a holistic methodological process for the assessment of skin quality, a dynamic and comprehensive skin quality scale (SQS) was developed that enables a holistic assessment of skin quality, helps identify the treatment priorities, facilitates conversation about skin quality with the subject and helps manage expectations, supports the creation of a long‐term treatment plan, and helps track treatment progress over time.

## 2 Methods

### 2.1 SQS Development

**FIGURE 1:**
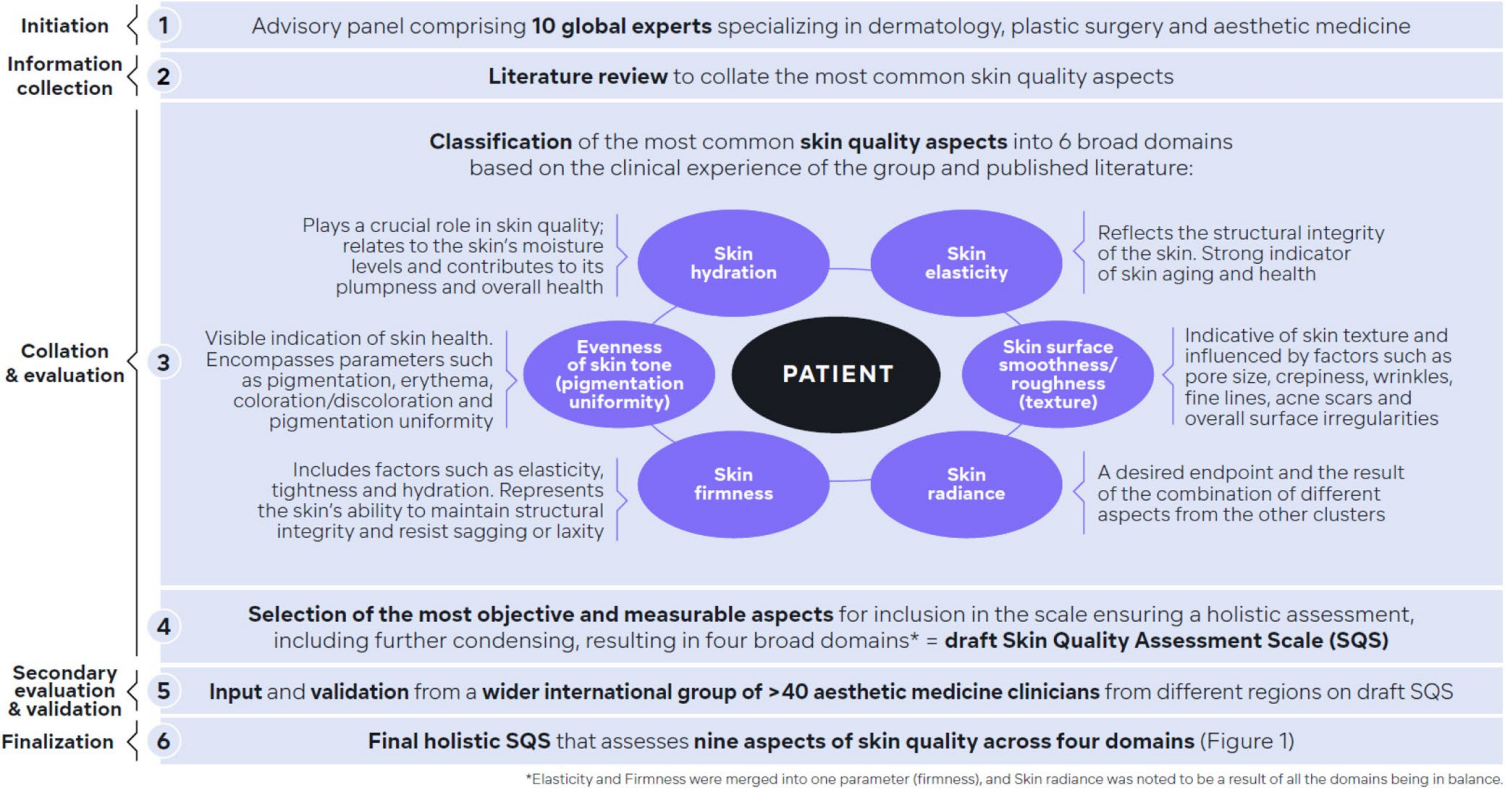
Development process of the Skin Quality Assessment Scale.

Global experts were selected on the basis that they specialized in dermatology and esthetic medicine, were based, within the group, across different continents to allow the SQS to have a global reach, and view skin quality as a critical patient concern in their clinics. From these criteria, a panel of 10 global experts across four continents, specializing in dermatology and esthetic medicine, developed the SQS in stages (see Figure 1).

#### 2.1.1 Information Collection

First, the expert panel collated the most common factors and measurements usually mentioned in the published literature. The multitude of sometimes overlapping terms used to evaluate the different aspects of skin quality illustrated the unresolved attempts to isolate the main skin quality markers.

#### 2.1.2 Collation and Evaluation

Then, the experts discussed how to best condense these different aspects of skin quality. This resulted in the clustering and classification of the most common aspects of skin quality and their measurability into six broad domains (Table 1) based on the clinical experience of the group and published literature. Each measurable aspect was evaluated by the expert group for its ability to assess and classify skin quality based on visual or tactile assessment or self‐assessment and information available in the published medical literature, without the need for specific assessment tools and thereby increasing the accessibility of the scale. The ranking of the six broad domains together with the rationale supporting this are shown in Table 1.

**TABLE 1:** Clustering measurable aspects of the skin and their ranking for potential inclusion in the SQS.

Ranking	Cluster	Rationale
1	Skin hydration	Plays a crucial role in skin quality, relates to the skin's moisture levels, and contributes to its plumpness and overall health
2	Elasticity	Reflects the structural integrity of the skin. This is the skin's ability to stretch and bounce back into position after stretching, which relies mostly on elastin fibers. Elasticity is a strong indicator of skin aging and health
3	Evenness of skin tone (pigmentation uniformity)	Visible indication of skin health. This encompasses measurable aspects, such as pigmentation, erythema, coloration/discoloration, and pigmentation uniformity
4	Skin surface smoothness/roughness (texture)	Indicative of skin texture and influenced by factors, such as pore size, crepiness, wrinkles, fine lines, acne scars, and overall surface irregularities
5	Skin firmness	Firmness is how tight the skin adheres to the underlying support (its tautness) provided by collagen and represents the skin's ability to maintain structural integrity providing shape and support and hence resist sagging or laxity
6	Skin radiance (glow)	Radiance represents the skin's ability to reflect light optimally, giving it a healthy glow. Specular reflection takes place from a smooth surface, such as a youthful skin or skin to which a moisturizer or makeup has been applied, while diffuse reflection takes place from a rough surface, such as textured skin with pores, fine lines, and wrinkles

**FIGURE 2:**
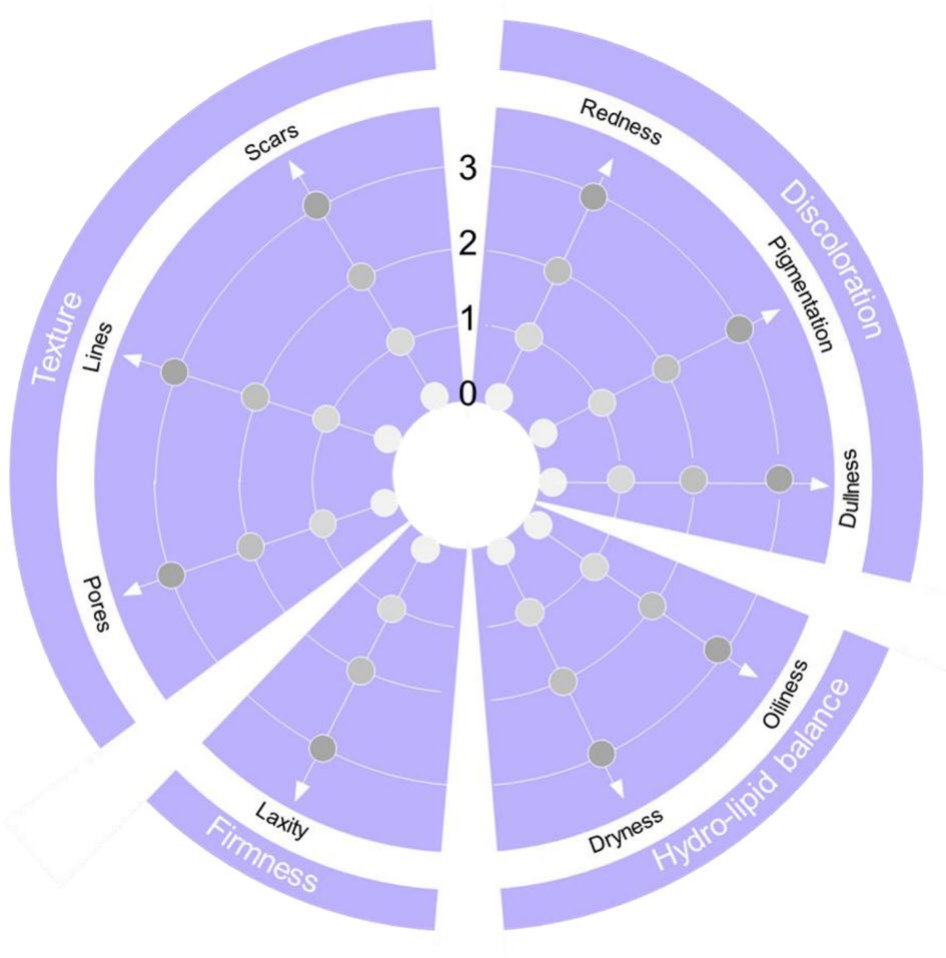
The Skin Quality Assessment Scale. Each aspect is graded on a 4‐point severity evaluation scale: 0 (none), 1 (mild), 2 (moderate), or 3 (severe), with the more severe scores on the outside of the chart.

Discussions among the expert panel took place on the different measurable aspects of skin quality within these six domains, together with the reproducibility of their measurement; the most objective and least variable measurable aspects of skin quality were chosen for inclusion in the SQS. This resulted in reducing these six domains down to four containing nine measurable aspects:

- Texture (Rank 4 in Table 1) assesses the measurable aspects of pore size, lines (crepiness or wrinkles), and (acne) scars (Figure 2).
- Discoloration assesses evenness of skin tone (Rank 3 in Table 1) and includes the measurable aspect of redness (erythema), which incorporates the presence of skin conditions such as acne or rosacea and entails an assessment of dyspigmentation and dullness (Figure 2).
- Firmness is the skin's ability to maintain structural integrity while remaining flexible; firmness (Rank 5 in Table 1) provides shape and support and in doing so it resists sagging or laxity, which is how it is assessed in the SQS (Figure 2).
- Hydro‐lipid balance is the balance of water and lipids in the skin; it has practical relevance and depending on whether skin is lacking water or lipids, different topical regimens will be applied. Hydration is a key measurable aspect (Rank 1 in Table 1) and hydro‐lipid balance is assessed in the SQS as levels of dryness and oiliness (Figure 2).

The terms elasticity, firmness, and laxity appear to often be used synonymously in the published literature. Based on this, the expert panel agreed that skin elasticity will be captured within the firmness (laxity) domain and is not included as a separate aspect of the SQS.

Skin radiance, or “glow” (Rank 6, Table 1), is the desired outcome of all the above measurable aspects being in balance and represents an overall measure of healthy, youthful‐looking skin; it is therefore not included as a separate measurable aspect of the SQS.

#### 2.1.3 SQS Creation

The SQS comprises four domains containing nine measurable aspects of skin quality (Figure 2):

- Texture (pores, lines, scars)
- Discoloration (redness, pigmentation, dullness)
- Firmness (laxity)
- Hydro‐lipid balance (oiliness, dryness)

Each aspect is graded on a 4‐point severity evaluation scale: 0 (none), 1 (mild), 2 (moderate), or 3 (severe).

The grades are assigned based on the expertise of the assessing physician and then discussed with the subject in a collaborative approach. The scores are plotted on a radar chart (a graphic that resembles a spider's web) (Figure 2), and the points are then joined to create a unique profile that “points toward” the subject's treatment priorities. The radar chart was chosen because of positive results using this type of chart for the assessment of subject satisfaction with facial treatment [24].

#### 2.1.4 Secondary Evaluation and Validation

The SQS was then shared for input and validation via an online survey (see supporting information for survey) to a wider group of > 40 esthetic medicine clinicians (comprising dermatologists, plastic surgeons, cosmetic physicians, primary care physicians, and nurses) from around the world. The survey enabled clinicians to validate the measurable aspects that contribute to skin quality, how skin quality is currently being assessed, the tools and methods currently being used and the utility of the SQS in clinical practice. Invited clinicians completed the survey between November 15, 2023 and January 9, 2024.

### 2.2 Real‐World Application of SQS

Clinicians involved in the development and validation of the SQS had the opportunity to use the tool and assign scores based on photographic and medical records from their own documented cases to evaluate and illustrate the practical applicability of the SQS. Informed consent was obtained from each person prior to treatment. The six case studies presented in this article illustrate the use and advantages of the SQS in clinical practice.

## 3 Results

### 3.1 Surveyed Group

In total, 42 practicing clinicians responded to the online survey on skin quality and the SQS. Most respondents were dermatologists (78%), with the remainder comprising plastic surgeons (5%), cosmetic physicians (5%), primary care physicians (5%), nurses (2%) or other (5%); 62% of respondents had been practicing esthetic medicine for over 10 years, 17% for 6–10 years, 10% for 3–6 years, and 11% for < 3 years. Two‐thirds of respondents (68%) assessed skin quality in subjects who came for treatment that was not specifically related to the skin.

### 3.2 The Need for a Holistic Skin Quality Assessment Tool

**FIGURE 3:**
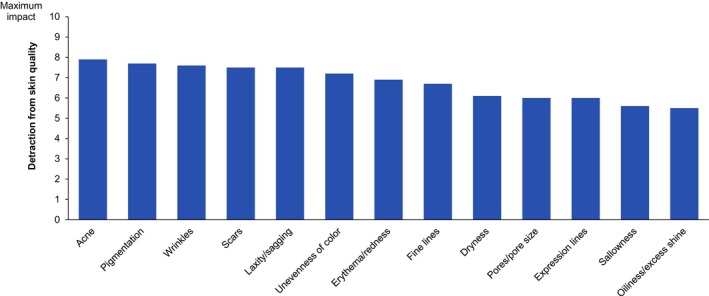
Mean values for detraction from skin quality (0 = no impact, 10 = maximum impact) for aspects related to the skin (*n* = 42).

According to the surveyed clinicians, a number of aspects can detract from skin quality, with acne, scars, wrinkles, laxity/sagging, pigmentation, and unevenness of color having the most impact (Figure 3).

**FIGURE 4:**
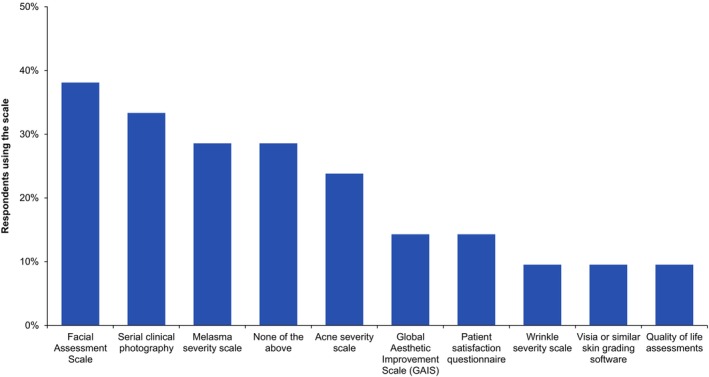
The percentage use of each scale in the subgroup of clinicians that used a scale to assess skin quality (*n* = 21) (note, a clinician could select more than one scale).

**FIGURE 5:**
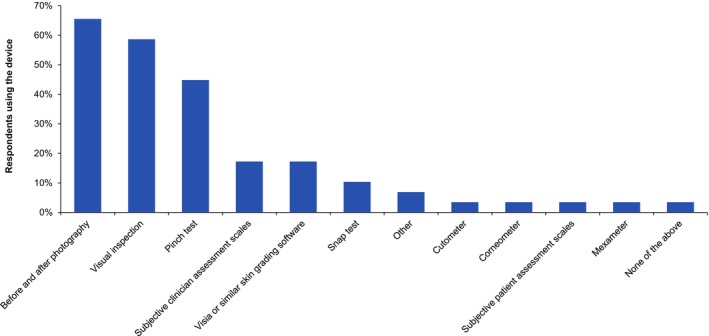
The percentage use for each device in the subgroup of clinicians that used a device to assess skin quality (*n* = 29) (note, a clinician could select more than one device).

Two‐thirds (67%) of clinicians did not use a scale to assess skin quality, and of those that did, there was no single specific scale in use (Figure 4). Of the 62% of clinicians that used a device to assess skin quality, before and after photography was most frequently used (by 66%), followed by the visual assessment (58%) and pinch test (45%) (Figure 5).

Almost all clinicians (96%) agreed that a collaborative approach where the clinician assigns severity grades and discusses these with the subject enhances the accuracy and relevance of an assessment scale. The majority (81%) did not believe that there was currently a holistic skin quality assessment tool that fulfills this need and is easy to use.

### 3.3 The SQS Provides a Holistic Skin Quality Assessment Tool for Clinicians

**FIGURE 6:**
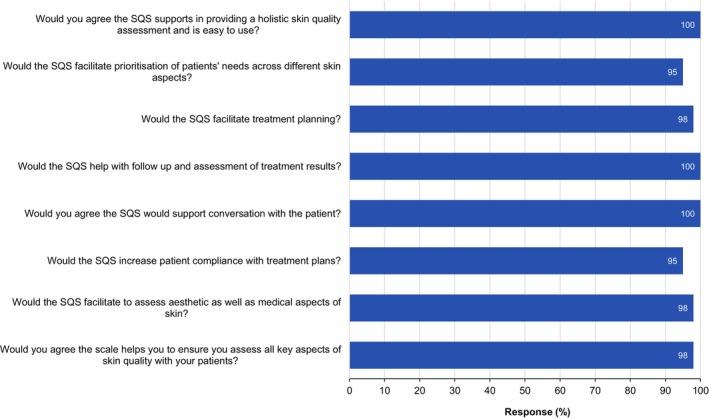
Clinicians' responses (combined percentage of those responding very much, much, and somewhat) to the questionnaire on the SQS (*n* = 42).

After a review of the SQS, 100% of respondents agreed that it supports providing a holistic skin quality assessment and is easy to use, 95% agreed that the scale helps to facilitate prioritization of patients' needs across different skin aspects, and 98% agreed that the SQS facilitates treatment planning and deemed it valuable for their clinic (Figure 6). Furthermore, 100% of respondents agreed that the SQS will help with follow‐up and assessment of treatment results. All respondents agreed the scale will support conversations with patients and 95% agreed it will increase patient compliance with treatment plans (Figure 6).

### 3.4 Practical Considerations: Real‐World Use of the SQS

The practical applicability of the SQS was evaluated in clinical practice and involved a number of patients, six of which are presented here.

**FIGURE 7:**
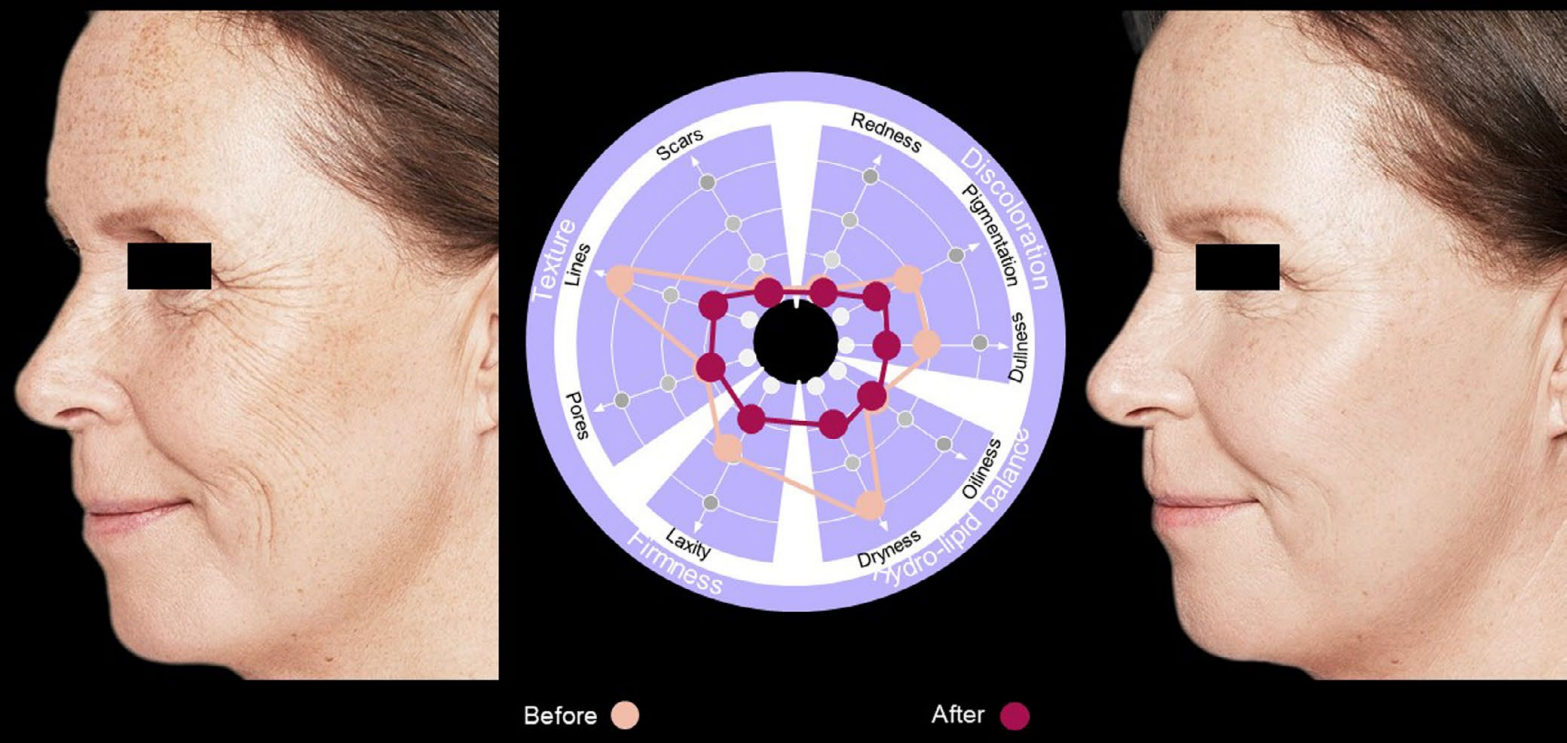
Case 1: Assessment using the SQS before and 90 days after treatment.

Case  1 For this 54‐year‐old female, using the SQS identified lines and dryness of the skin as the areas of concern, followed by pigmentation and dullness (Figure 7). Reassessment 5 months after treatment showed improvements in all the identified aspects and that of laxity (Figure 7).

**FIGURE 8:**
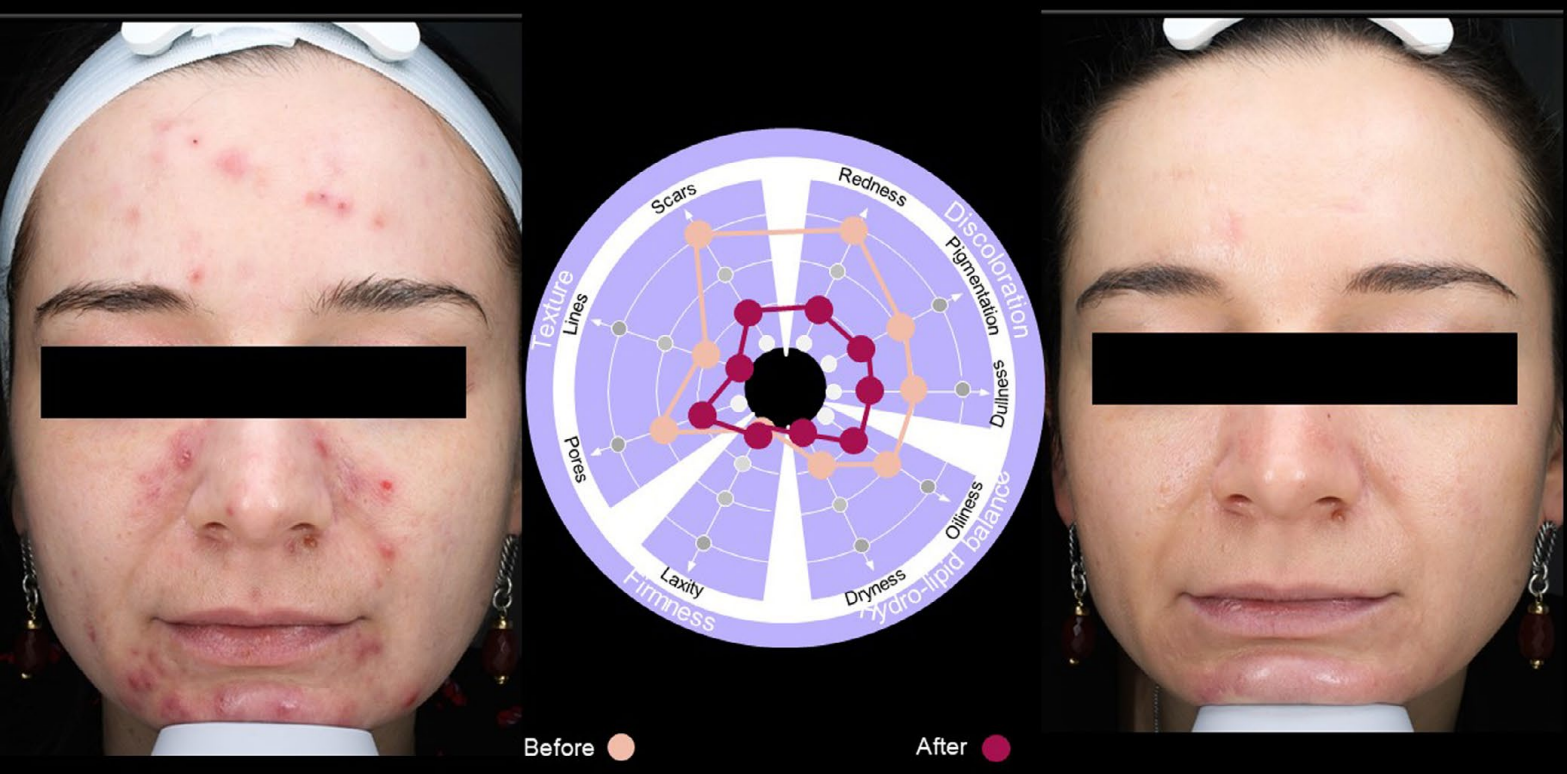
Case 2: Assessment using the SQS before and 90 days after treatment.

Case  2 For this 37‐year‐old female (Figure 8), the use of the SQS identified particular issues with scars and redness, as well as problems with pigmentation, dullness, oiliness, and pore size. Reassessment using the SQS 90 days after treatment demonstrated improvement in all skin quality domains, which could be clearly seen in before and after photographs (Figure 8).

**FIGURE 9:**
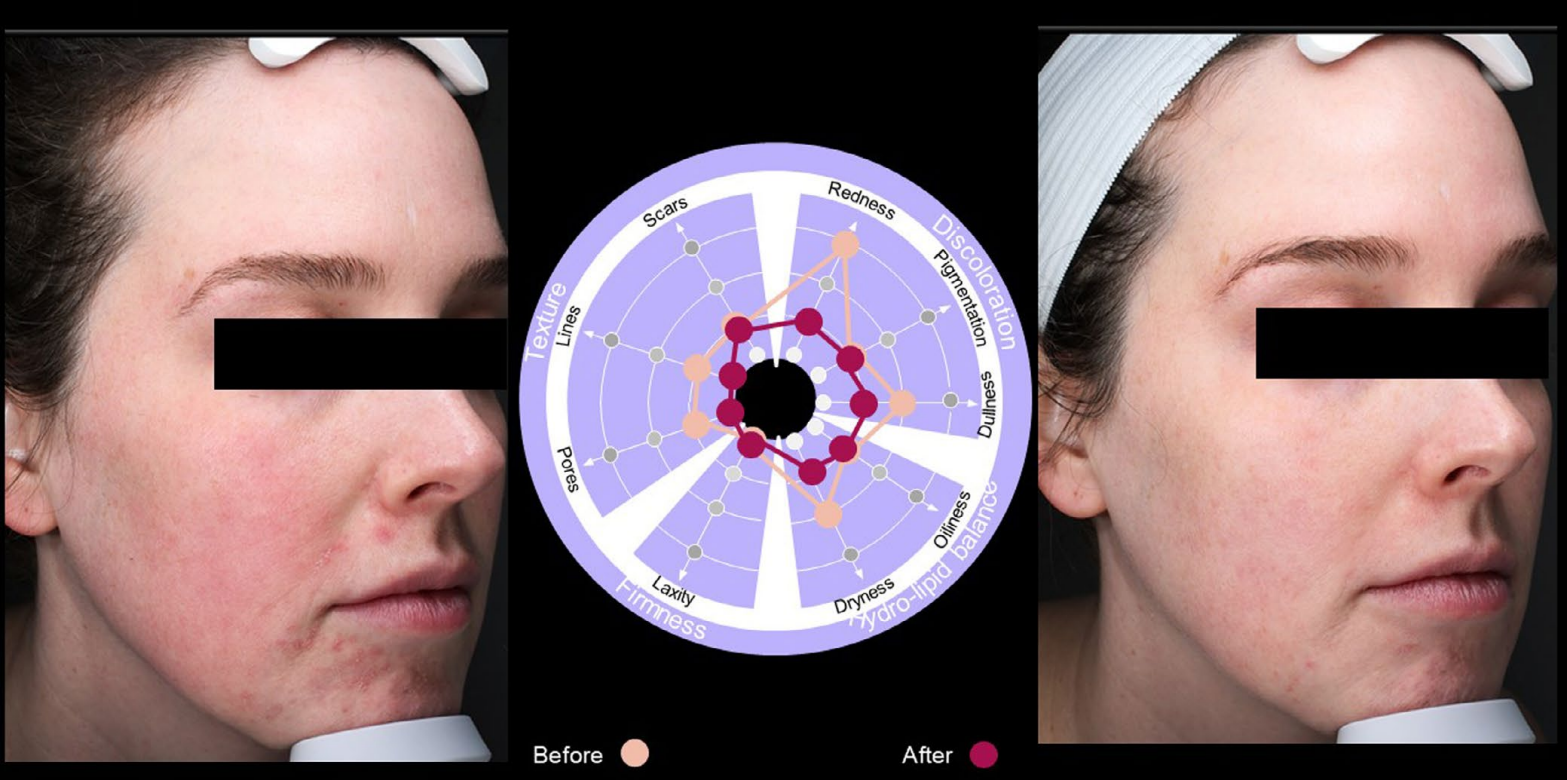
Case 3: Assessment using the SQS before and 90 days after treatment.

Case  3 Use of the SQS with this 34‐year‐old female (Figure 9) identified particular concerns regarding redness and issues with dullness and dryness of the skin. Reassessment 90 days after treatment using the SQS showed substantial improvement in redness and improvement in dullness and dryness, for a more balanced skin quality profile (Figure 9).

**FIGURE 10:**
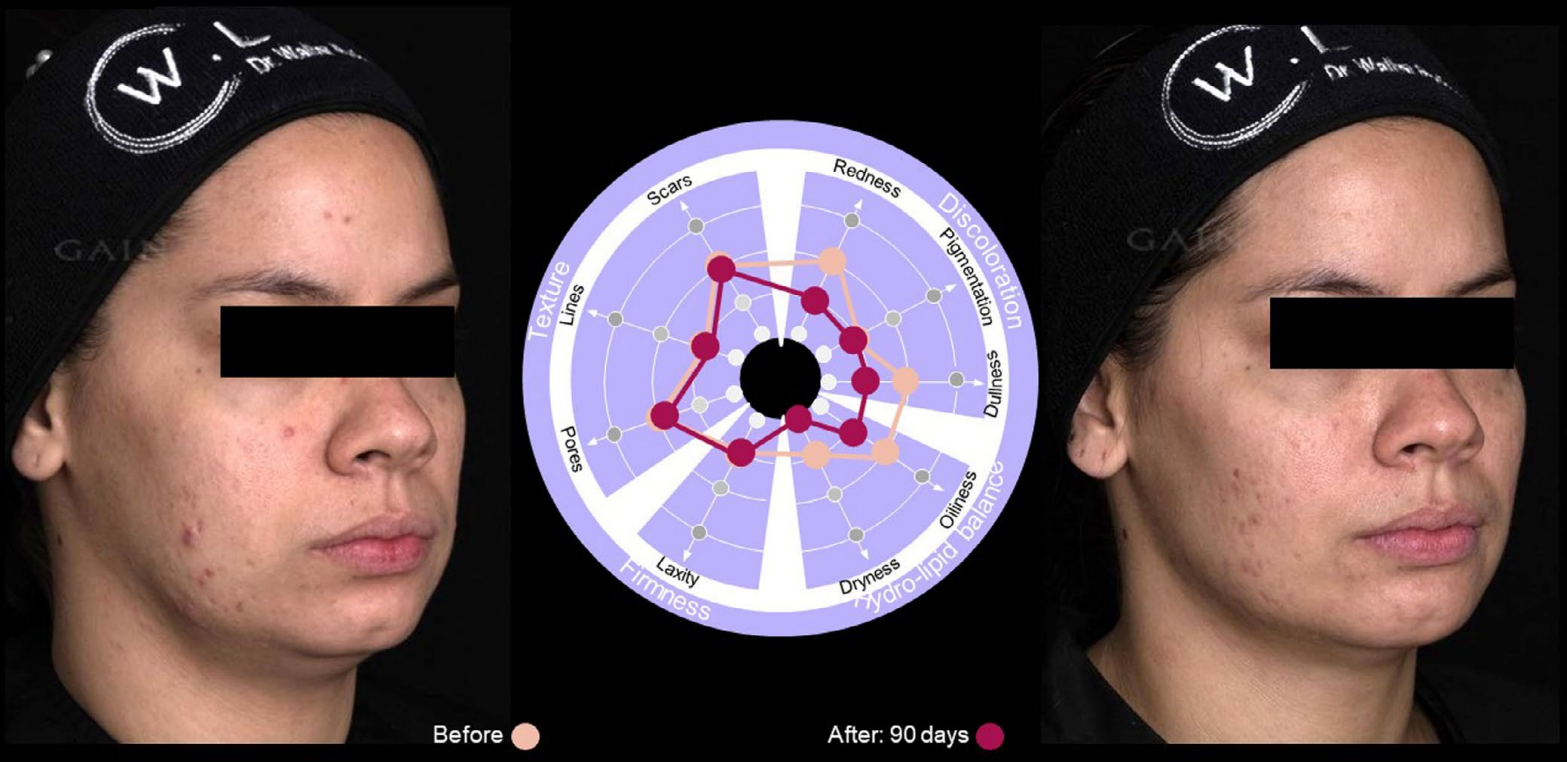
Case 4: Assessment using the SQS before and 90 days after treatment.

Case  4 In this 46‐year‐old female, the use of the SQS identified particular concerns with redness, dullness, oiliness, and visible pores and scars (Figure 10). Reassessment using the SQS 90 days after treatment demonstrated improvements in redness, dullness, and oiliness (Figure 10).

**FIGURE 11:**
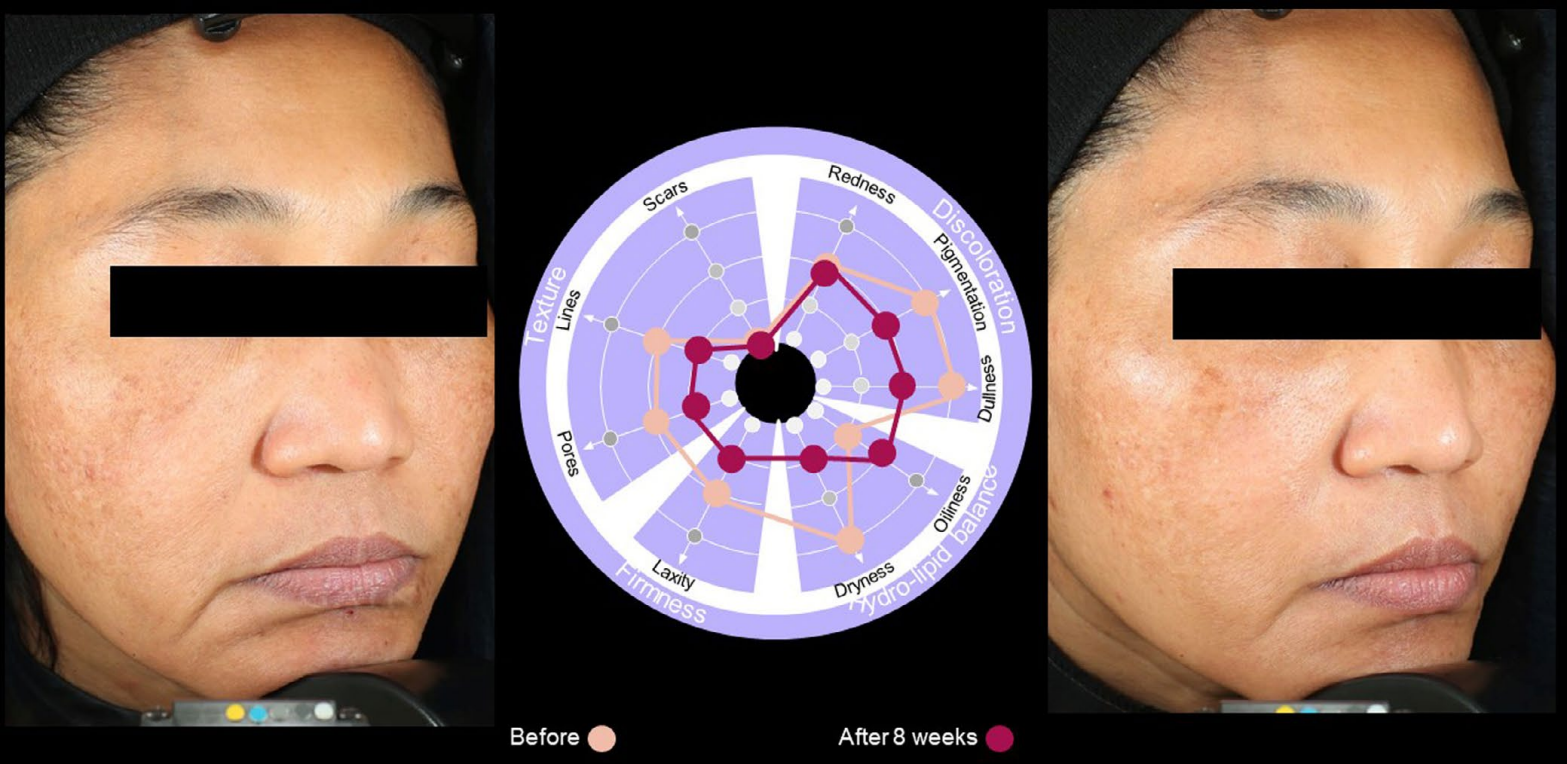
Case 5: Assessment using the SQS before and 8 weeks after treatment.

Case  5 In this 43‐year‐old female, the SQS identified treatment priorities of pigmentation, dullness, and dryness, with laxity, pores, and lines also being a concern. Eight weeks after treatment, use of the SQS indicated particular improvements being observed in pigmentation, dullness, and dryness of the skin (Figure 11).

**FIGURE 12:**
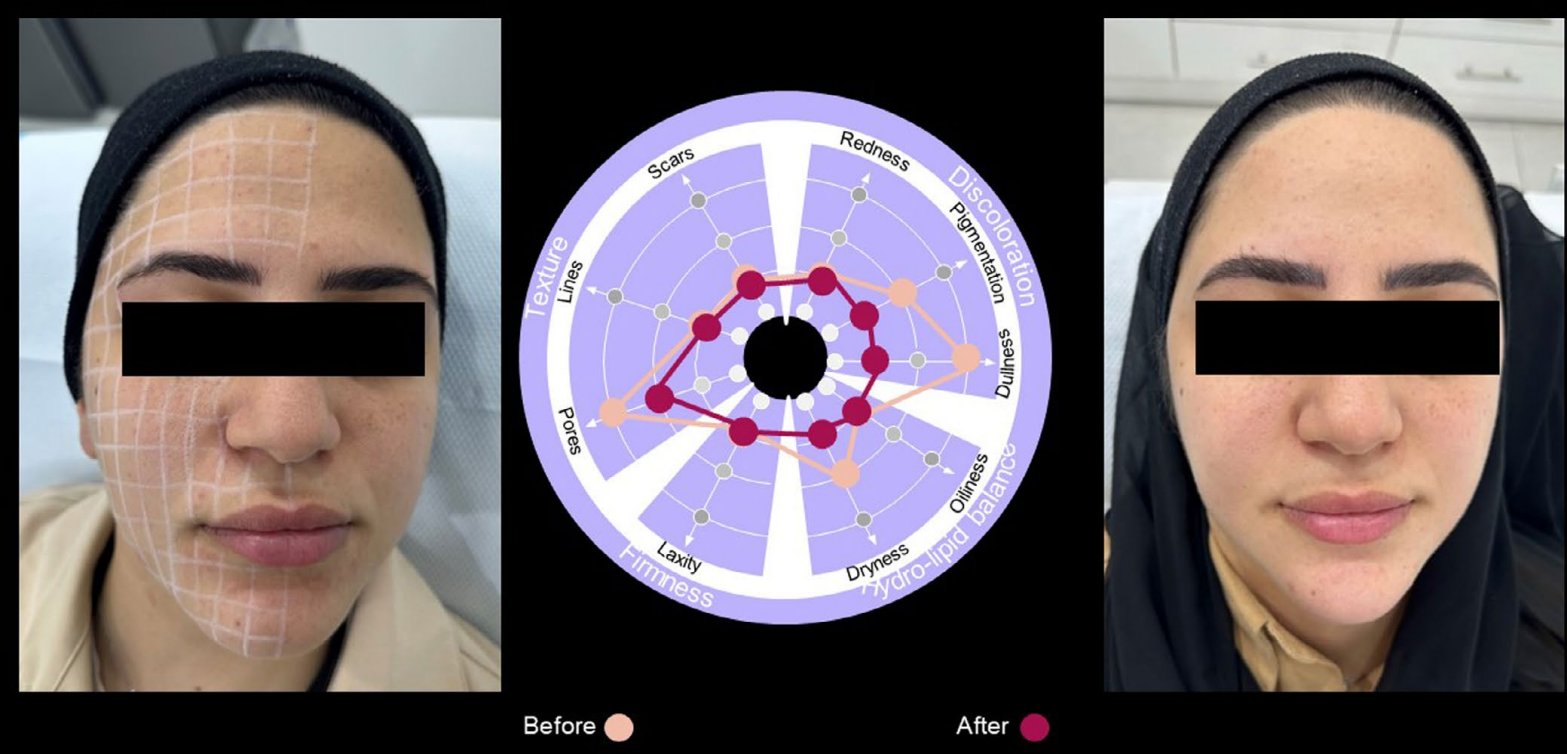
Case 6: Assessment using the SQS before and 6 weeks after treatment.

Case  6 In this 29‐year‐old female, use of the SQS identified particular issues with dullness and pores, for which the SQS scores all improved 6 weeks after treatment (Figure 12).

## 4 Discussion

The need for a methodological process that evaluates skin quality from a holistic approach prompted the development of the SQS. The prior lack of a comprehensive holistic scale was highlighted by 81% of surveyed clinicians, who did not believe that there was currently a skin quality assessment tool that fulfills this need.

By including four domains that contain nine measurable aspects that are each assessed using a 4‐point severity scale, the SQS is the first scale that allows a comprehensive holistic assessment of skin quality. In addition, use of the SQS engages with and manages subjects' expectations, is easily accessible to all treating physicians, identifies the treatment priorities (by “pointing” in a certain direction), creates a long‐term treatment plan, and, by evaluation post‐treatment, visualizes the skin quality improvement over time. Moreover, the SQS is designed to be convenient and support a collaborative approach where the physician assigns severity grades to the measurable aspects and discusses these with the subject to align on treatment expectations.

The comprehensive and holistic nature of the SQS means that it assesses many aspects of skin quality rather than an individual aspect in depth, which could be considered a limitation. However, the use of the SQS for an initial assessment to identify any specific treatment priorities or areas of concern can always be further evaluated in more detail using a specific scale, such as evaluation of acne scars [21], rosacea [22], skin texture, and homogenous pigmentation [1]. Assessment of the SQS by > 40 surveyed clinicians found that 100% agreed the scale provides a holistic skin quality assessment and 95% agreed that the SQS helps to ensure that clinicians assess all key aspects of skin quality with their subjects. Almost all respondents (98%) agreed that the SQS facilitates treatment planning and deemed it valuable for their clinic, and 100% agreed that the scale will help with follow‐up and assessment of treatment results. The importance of a collaborative approach was supported by all surveyed clinicians who agreed that the SQS would support conversation with the subject.

The SQS represents the first step in the assessment of the subject's skin quality. Evaluation of the SQS in real‐world clinical practice, illustrated in this article by six case studies, indicates that its use before treatment will help to determine the immediate concerns of the subject and its use post‐treatment will help demonstrate the level of success following treatment. An additional benefit is that the use of the SQS post‐treatment can open further conversation regarding potentially secondary concerns and support the creation of a long‐term treatment plan. The continued use of the SQS in clinical real‐world practice will help to further refine this new methodological approach to skin quality assessment.

## Author Contributions

All authors provided input into the design, evaluation, interpretation, and validation of the skin quality assessment scale, and in the development of this manuscript. C.M. led the development of both the scale and the manuscript. All authors reviewed each version of the manuscript and approved its submission to the journal.

## Ethics Statement

The authors have nothing to report.

## Consent

All subjects provided signed consent for their images to be used.

## Conflicts of Interest

Christoph Martschin is a speaker, trainer, consultant, and clinical trial investigator for Galderma. Ruba Bahhady is a speaker, trainer, consultant, and clinical trial investigator for Galderma. Jason Li is a speaker, trainer, consultant, and clinical trial investigator for Galderma. Walter Loureiro is a speaker, trainer, consultant, and clinical trial investigator for Galderma. Wesam Mansour is a speaker, trainer, consultant, and clinical trial investigator for Galderma. Andrei Metelitsa is a speaker, trainer, consultant, and clinical trial investigator for Galderma. Kuldeep Minocha is a speaker, trainer, consultant, and clinical trial investigator for Galderma. Michael Somenek is a speaker, trainer, consultant, and clinical trial investigator for Galderma. Keywan Taghetchian is a speaker, trainer, consultant, and clinical trial investigator for Galderma. Tanongkiet Tienthavorn is a speaker, trainer, consultant, and clinical trial investigator for Galderma. Medical writing assistance was provided by Dr. Ian Wright, PhD, on behalf of Galderma.

## Supporting information


Data S1.

## Data Availability

The data that support the findings of this study are available from the corresponding author upon reasonable request.
