# Supplementary material for: Development and Validation of a Novel Holistic Skin Quality Assessment Scale
Source: J Cosmet Dermatol. 2024 Oct 9;24(1):e16615. doi: 10.1111/jocd.16615 (PMC11743243; doi:10.1111/jocd.16615)
Supplement: Supplementary file 1 — Data S1. [file JOCD-24-e16615-s001.docx]

**Supplementary material**

**Skin Quality Assessment Scale Survey**

### **Overview**

Multiple factors contribute to skin quality and there are existing scales/measures that focus on different aspects of skin quality, such as acne scars, rosacea, skin texture and homogenous pigmentation, and scales that focus on specific aspects, such as the Wrinkle Severity Rating Scale.

Our objective was to approach the assessment of skin quality from a holistic perspective and to develop a simple method that would help physicians to assess skin quality and the underlying elements of skin quality. It was also considered important that this novel skin quality assessment scale could be used by patients to allow both parties to track the patient’s journey together and aid clinician–patient discussions.

To this end, a skin quality Scientific Exchange Focus Group comprising a global panel of dermatologists and aesthetic physicians identified the main factors/parameters that define optimal skin quality. This survey has been designed to gain your insights into these factors and their importance. The responses will be used to facilitate discussion and utilize appropriate factors in the development of a skin quality assessment scale. This will help its practical application and utility in clinical practice.

Thank you in advance for your input and contribution.

### SURVEY QUESTIONS

| 1. What is your primary medical specialty? | **[Select one only]**   - Dermatologist - Surgeon (Plastic/Cosmetic) - Oral maxillofacial surgeon - Oculoplastic surgeon - Cosmetic physician - Primary care physician/General Practitioner - Registered Nurse/Nurse Practitioner - Other |
| --- | --- |
| 1. How many years have you been practicing aesthetic medicine? | **[Select one only]**   - <12 months - 1-3 years - 3-5 years - 5-10 years - >10 years |
| 1. Do you measure/assess skin quality in patients who come for unrelated treatments? | **[Select one only]**   - No - Yes |
| 1. To what extent does unevenness of color **detract** from optimal skin quality? | **[Mark the most appropriate number on the scale;  *0 = no impact, 10 = maximum impact*]** |
| 1. To what extent does erythema/redness **detract** from optimal skin quality? | **[Mark the most appropriate number on the scale;  *0 = no impact, 10 = maximum impact*]** |
| 1. To what extent does pigmentation **detract** from optimal skin quality? | **[Mark the most appropriate number on the scale;  *0 = no impact, 10 = maximum impact*]** |
| 1. To what extent does sallowness/yellow undertone **detract** from optimal skin quality? | **[Mark the most appropriate number on the scale;  *0 = no impact, 10 = maximum impact*]** |
| 1. To what extent does dryness **detract** from optimal skin quality? | **[Mark the most appropriate number on the scale;  *0 = no impact, 10 = maximum impact*]** |
| 1. To what extent does oiliness/excess shine **detract** from optimal skin quality? | **[Mark the most appropriate number on the scale;  *0 = no impact, 10 = maximum impact*]** |
| 1. To what extent does pores/pore size **detract** from optimal skin quality? | **[Mark the most appropriate number on the scale;  *0 = no impact, 10 = maximum impact*]** |
| 1. To what extent does fine lines **detract** from optimal skin quality? | **[Mark the most appropriate number on the scale;  *0 = no impact, 10 = maximum impact*]** |
| 1. To what extent does wrinkles **detract** from optimal skin quality? | **[Mark the most appropriate number on the scale;  *0 = no impact, 10 = maximum impact*]** |
| 1. To what extent does expression lines **detract** from optimal skin quality? | **[Mark the most appropriate number on the scale;  *0 = no impact, 10 = maximum impact*]** |
| 1. To what extent does acne **detract** from optimal skin quality? | **[Mark the most appropriate number on the scale;  *0 = no impact, 10 = maximum impact*]** |
| 1. To what extent does scars **detract** from optimal skin quality? | **[Mark the most appropriate number on the scale;  *0 = no impact, 10 = maximum impact*]** |
| 1. To what extent does laxity/sagging **detract** from optimal skin quality? | **[Mark the most appropriate number on the scale;  *0 = no impact, 10 = maximum impact*]** |
| 1. Do you use a scale to measure/assess your patients’ overall skin quality? | **[Select one only]**   - No - Yes |
| 1. If yes, which of the following scales do you use in your practice? | **[Select all that apply]**   - Facial Assessment Scale - Acne severity scale - Melasma severity scale - Wrinkle severity scale - Global aesthetic improvement scale - Patient satisfaction questionnaire - Photo-numeric scales - Visia or similar skin grading software - Serial clinical photography - Quality of life assessments - Other [Please state] - None of the above |
| 1. Do you use any devices to measure/assess your patients’ overall skin quality? | **[Select one only]**   - No - Yes |
| 1. If yes, what devices are you using to measure/assess your patients’ overall skin quality? | **[Select all that apply]**   - Cutometer - Snap Test - Pinch test - Before and after photography - Corneometer - Subjective clinician assessment scales - Subjective patient assessment scales - Mexameter - Visia or similar skin grading software - Visual inspection - Other [Please state] - None of the above |
| 1. Do you believe that a collaborative approach, where both the physician and patient allocate grades, enhances the accuracy and relevance of the assessment? | **[Select one only]**   - No - Yes |
| 1. Is there currently any scale that is easy to use and that fulfill this need? | **[Select one only]**   - No - Yes |

**Skin Quality Assessment Scale**

Multiple factors contribute to skin quality and there are existing scales/measures that focus on different aspects of skin quality. We want to approach the assessment of skin quality from a holistic perspective and develop a simple method that would help physicians to assess skin quality and the underlying elements. It was also considered important that this novel skin quality assessment scale could be used with patients to allow both parties to track the patient’s journey together and aid clinician–patient discussions.


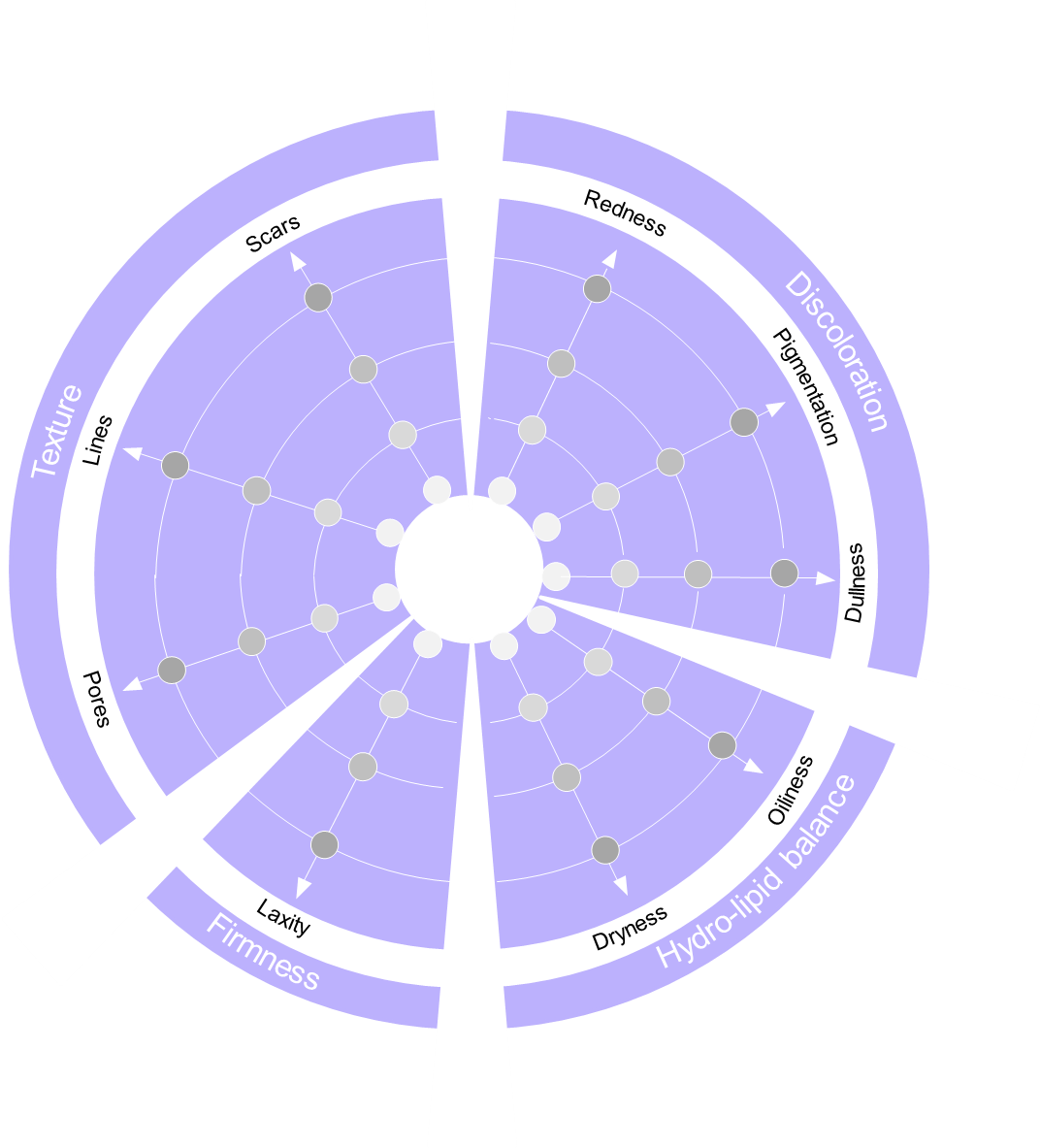


| 1. In your opinion does the suggested SQS fulfil this need? | **[Select one only]**   - Very much - Much - Somehow - No |
| --- | --- |
| 1. Would the SQS facilitate prioritisation of patients’ needs across different skin aspects? | **[Select one only]**   - Very much - Much - Somehow - No |
| 1. Would the SQS facilitate to assess aesthetic as well as medical aspects of skin? | **[Select one only]**   - Very much - Much - Somehow - No |
| 1. Would the SQS facilitate treatment planning? | **[Select one only]**   - Very much - Much - Somehow - No |
| 1. Would the SQS increase patient compliance with treatment plans? | **[Select one only]**   - Very much - Much - Somehow - No |
| 1. Would the SQS help with follow up and assessment of treatment results? | **[Select one only]**   - Very much - Much - Somehow - No |
| 1. Would you agree the SQS supports in providing a holistic skin quality assessment? | **[Select one only]**   - Very much - Much - Somehow - No |
| 1. Would you agree the SQS would support conversation with the patient? | **[Select one only]**   - Very much - Much - Somehow - No |
| 1. Would you agree the scale helps you to ensure you assess all key aspects of skin quality with your patients? | **[Select one only]**   - Very much - Much - Somehow - No |
| 1. If not, what would you want to add/which parameter(s) do you believe is missing? | **Open answer** |
